# Safety of Intraoperative Cell Salvage in Two-Stage Revision of Septic Hip Arthroplasties

**DOI:** 10.3390/antibiotics13090902

**Published:** 2024-09-21

**Authors:** Lara Krüger, André Strahl, Eva Goedecke, Maximilian M. Delsmann, Leon-Gordian Leonhardt, Frank Timo Beil, Jan Hubert

**Affiliations:** Division of Orthopaedics, Department of Trauma and Orthopaedic Surgery, University Medical Centre Hamburg-Eppendorf, 20246 Hamburg, Germany

**Keywords:** periprosthetic joint infection, cell salvage, patient blood management, septic surgery, revision arthroplasty

## Abstract

(1) Background: The aim of this study was to evaluate the safety of intraoperative cell salvage (ICS) during reimplantation in the two-stage revision of septic hip arthroplasties. (2) Methods: As part of an internal quality control study, blood cultures were taken from the processed ICS blood during reimplantation and examined for possible bacterial load (study group). Due to a high rate of bacterial detection with uncertain clinical significance, consecutive ICS samples were also examined from patients undergoing aseptic revision hip arthroplasty (control group). Microbiological samples, patient and surgical characteristics and the follow-up data were analyzed retrospectively. (3) Results: 9 out of 12 (75%) patients in the study group and 5 out of 8 (63%) patients in the control group had positive ICS blood cultures. There was no significant difference between the groups (*p* = 0.642). The initial pathogens causing the periprosthetic joint infection (PJI) were not detected, but the bacterial spectrum resembled skin flora, with a high proportion of coagulase-negative staphylococci. No complications due to possible bloodstream-associated infections were observed. In summary, the detected pathogens were interpreted as contamination without clinical significance. (4) Conclusions: ICS in the context of reimplantation was considered a safe and recommendable procedure to optimize patient blood management.

## 1. Introduction

The incidence of joint replacement surgery is increasing as life expectancy rises and the population ages due to demographic changes. In this context, both aseptic and septic revision procedures are being performed more frequently [1]. In particular, periprosthetic joint infections (PJI) pose a specific challenge for the treating surgeon as they are among the most severe complications of joint replacement surgery. PJIs are associated with markedly high morbidity and mortality [2] and require a comprehensive treatment concept, which often involves complex revision surgery. The two-stage procedure, which includes the initial removal of the prosthesis, subsequent targeted antibiotic therapy and reimplantation arthroplasty at a later stage, has proven to be an effective treatment approach [3]. However, compared to traumatic and aseptic hip arthroplasties, septic revision surgeries have been associated with the highest average blood loss [4] and often require the transfusion of blood products to restore an adequate hemoglobin level.

The transfusion of allogeneic packed red blood cells (pRBCs) is associated with various potential complications and carries the risk of a reduced outcome. For example, transfusion-related immune modulation has been linked to a significantly higher rate of infections, such as pneumonia and surgical site infections [5,6,7]. Additional relevant risks include volume and electrolyte shifts, anaphylactic reactions, infections caused by pathogen-contaminated blood products and transfusion incidents such as patient mix-ups [8,9].

Patient blood management represents a central element in the prevention of allogeneic pRBC transfusions and the associated complications. Intraoperative cell salvage (ICS), which involves collecting the patient’s own blood for re-transfusion after processing, has proven to be one of the most effective measures in avoiding allogeneic pRBC transfusions [8,10,11,12]. If increased blood loss is anticipated during surgery, the use of ICS is recommended in accordance with current anesthesiology guidelines [13]. Particularly for patients undergoing hip revision surgery, ICS has been shown to be effective in reducing the rate and volume of allogeneic pRBC transfusions [8].

The scientific guidelines on the use of ICS in the presence of surgical site infections (SSI) are inconclusive. Formally, there are no absolute contraindications against the use of ICS [13]. However, an infection at the surgical site was described as a relative contraindication, depending on the likelihood/degree of contamination [13]. Even though an SSI could potentially lead to bacterial contamination of the salvaged blood and subsequent systemic spreading of pathogens, there is no conclusive evidence that ICS worsens sepsis, prognosis or the risk of other specific complications when used in the contaminated field [13,14].

Based on positive clinical experience, it has been common practice in the past to use ICS during two-stage reimplantation, assuming that the PJI had been sufficiently treated [4]. However, a consensus statement or comprehensive scientific literature with treatment recommendations does not currently exist [15]. A recent study of 144 patients who received ICS blood as a part of the reimplantation procedure found no accumulation of septic complications in the short-term or long-term follow-up [16]. However, whether there is a detectable persistent load of PJI-causing pathogens in the ICS blood has not yet been investigated.

This raises the following research questions for this study:

(1) Is the PJI-causing pathogen still detectable in the processed ICS blood during reimplantation after septic two-stage hip revision arthroplasty?
(2) Do patients who receive ICS blood as a part of the reimplantation procedure have a higher rate of septic complications compared to patients undergoing aseptic revision arthroplasty?

Therefore, the aim of this study was to further evaluate the safety of ICS in the context of reimplantation during septic two-stage hip revision surgery, contributing to the optimization of blood management for these critically ill patients.

## 2. Results

In 12 patients, one aerobic and one anaerobic blood culture from the processed ICS blood were collected during reimplantation in the context of septic two-stage hip revision arthroplasty (study group). An initial analysis revealed a high rate of microorganism-positive cultures, but it was unknown whether these were true infections or contaminations. To differentiate, ICS blood cultures were subsequently also taken from eight patients undergoing aseptic hip revision arthroplasty (control group), as no true infections were to be expected here. Due to the continued high rates of bacteria-positive samples also in the aseptic control group, which showed no recognizable clinical relevance, the sample collection was subsequently terminated to avoid unnecessary blood loss. In total, this resulted in a study population of 20 patients. The patient and surgery characteristics, results of the ICS blood cultures and the evaluation of possible signs of sepsis in the early postoperative short-term follow-up are listed in Table 1.

### 2.1. Patient and Surgical Characteristics

Except for the total number of allogeneic blood units transfused, which was significantly higher in the study group (*p* = 0.022), no significant differences were found between the patient and surgery characteristics of the study and control groups (Table 1).

### 2.2. ICS Blood Culture Results

In the study group, positive ICS blood cultures were found in 9 out of 12 (75%) patients (Table 1). In eight cases, only one culture medium was positive, while in one patient, both the aerobic and the anaerobic cultures showed positive results. In six cases, the positive ICS cultures were different from the pathogen that initially caused the PJI. The three identical pathogens included coagulase-negative staphylococci (*Staph. epidermidis*) in two cases and *Cutibacterium acnes* in one case. Due to the differences in the antimicrobial susceptibility patterns of the ICS and PJI pathogens, it was concluded that the ICS germs were different bacterial strains and not the bacteria that had initially caused the PJI; therefore, they were considered contaminants.

In the control group, five out of eight patients (63%) showed positive ICS blood cultures (Table 1). In four cases, only one culture was positive, while in one patient, both the aerobic and the anaerobic cultures showed positive results.

There was no significant difference between the rate of positive ICS cultures in the study and control groups (*p* = 0.642). In total, positive cultures were detected in the ICS blood of 14 out of 20 patients (70%) across both groups.

The types of pathogens found in the positive ICS cultures, categorized by study and control group, are shown in Table 2.

In addition, the five intraoperative tissue samples that were routinely taken during the reimplantation (study group) or aseptic revision surgery (control group) were analyzed. In one patient from the study group and one patient from the control group, one out of five samples showed a positive microbiological result: in one case, *Staphylococcus epidermidis* (study group), and in the other case, *Cutibacterium acnes* (control group).

Due to the detection in only one of five samples, contamination without clinical relevance was assumed, as is customary in clinical practice.

Additionally, histological investigations of the periprosthetic membranes were conducted according to the Krenn classification. The results, subdivided into patients with positive and negative ICS cultures, are shown in Table 3.

### 2.3. Follow-Up

In the early postoperative short-term follow-up, no higher incidence of possible signs of sepsis were observed in either patient group (Table 1).

The intermediate follow-up during the inpatient stay and the subsequent stay in the rehabilitation clinic averaged 37.6 ± 13.2 days (range: 18–73). No complications due to possible bloodstream-associated infections, such as endocarditis or spondylodiscitis, were observed in both groups.

The long-term follow-up averaged 19.2 ± 12.5 months (range: 11–39). Again, neither signs of possible bloodstream-associated infections nor recurrent periprosthetic or medical-device-associated infections were observed in both groups.

## 3. Discussion

Periprosthetic joint infections are among the most serious complications of arthroplastic surgery, and their optimal treatment still remains challenging for orthopedic surgeons today [17]. Adequate patient blood management, especially through the use of ICS, has the potential to effectively avoid complications by reducing the need for allogeneic pRBC transfusions [4]. Formally, an infection of the surgical field poses a contraindication against the use of ICS [13,14]. However, in the context of reimplantation after a two-stage septic revision arthroplasty, the use of ICS is a common clinical practice, although no consensus statement or comprehensive scientific literature with treatment recommendations exists [15]. Whether a persistent presence of the PJI-causing pathogens in the ICS blood can be detected during reimplantation has not yet been investigated.

Therefore, the aim of this study was to investigate the safety of ICS in the context of reimplantation in order to contribute to the optimization of therapy for the often critically ill PJI patients. This is the first study to examine the collected and processed ICS blood for a possible bacterial contamination in the context of septic or aseptic hip revision arthroplasties.

### 3.1. Patient and Surgical Characteristics

The patient and surgical characteristics of the study and control groups were compared. Apart from a higher rate of allogeneic pRBC transfusions in the study group (*p* = 0.022), there were no other significant differences, indicating that adequate comparability between the groups could be assumed while no confounding factors were detected. Our results are consistent with the previously published literature, which has described the frequently increased need of allogeneic pRBC transfusions during septic revisions compared to traumatic arthroplasties or aseptic revision arthroplasties [4].

### 3.2. Microbiological and Histological Findings

In order to provide the best possible growth conditions for all types of microorganisms, an aerobic and an anaerobic blood culture were taken from the processed ICS blood. All microbiological samples were consistently incubated for 14 days to enable the detection of low-grade infections, which may play a relevant role in PJIs [18]. By working under strictly sterile conditions with the setup of a separate sterile table, accidental contamination during sample processing was minimized.

Surprisingly, microorganisms were detected in the ICS blood in 75% of cases in the study group and in 63% in the control group. There was no significant difference in the frequency of positive ICS cultures between the groups (*p* = 0.642).

To this day, blood culture continues to be the gold standard for detecting possible bacteremia in everyday clinical practice [19]. However, as is often the case with highly sensitive tests, there is a risk of false-positive results that detect microorganisms with no clinical relevance [19]. Differentiating between true bacteremia and contamination can be a challenge even for the experienced clinician. Possible aids to help with differentiation include the type of organism detected, comparison of the antimicrobial susceptibility patterns, the number of positive cultures within a set and the “time to positivity,” which refers to the time it takes for an organism to grow in the culture medium [19].

The type of microorganism detected has been described by various authors as the most important aspect of blood culture assessment [19,20,21]. Weinstein et al. even stated that certain organisms, such as coagulase-negative staphylococci (e.g., *Staph. epidermidis*, *Staph. haemolyticus*, *Staph. capitis*), *Cutibacterium acnes* or *Bacillus species other than Bacillus anthracis*, are highly likely to be contaminations [22], whereas others, such as *Staphylococcus aureus* or *Candida species*, frequently indicate a true infection [23]. Coagulase-negative staphylococci, in particular, are the most common blood culture contaminants, accounting for 70–80% of all contaminated blood cultures [19,21,24,25,26]. At the same time, however, they are increasingly reported in the context of prosthetic and medical-device-associated infections [19,27,28]. However, a positive staphylococcal blood culture is reported to be a contamination in 73–74% of cases and correspondingly a true bacteremia in 26–27% [26,29]. The bacterial spectrum found in the ICS blood corresponded closely to the resident skin flora, with a high ratio of coagulase-negative staphylococci and Cutibacteria. Consequently, based on the literature, contamination can be assumed to be more likely.

However, the detection of *Staph. aureus* and *Candida species* had to be assessed together with other parameters. Therefore, the ICS samples were compared with the initial PJI microorganisms from the study group. In six cases, the initial PJI microorganism and the ICS culture were not identical; in three cases, the same organisms were detected (2x *Staph. epidermidis* and 1x *Cutibacterium acnes*). For a more precise assessment of the identified congruent organisms, the antimicrobial susceptibility patterns were compared. Due to the differences found, it was assumed that different bacterial strains were present and that the ICS findings were therefore contaminations. Since molecular testing of the isolated cultures is not feasible in everyday clinical practice, a comparison of the antimicrobial susceptibility patterns is commonly used to assess whether the isolates represent identical bacterial strains [19,29,30]. However, it should be critically noted that coagulase-negative species have the potential to cause polyclonal infections [31,32,33], so the assessment of the antimicrobial susceptibility patterns should always be performed in conjunction with other parameters.

Furthermore, the ICS samples were compared with all tissue samples that had been taken intraoperatively as a part of the reimplantation (study group) or aseptic revision surgery (control group). In both cases observed, only one of five samples was positive (1x *Staph. epidermidis*, 1x *Cutibacterium acnes*), which is commonly considered a contamination in everyday clinical practice. The scientific literature also agrees that the number of positive samples/bottles within a set is a valuable aid in differentiating between true bacteremia and contamination [19,22,23,34]. In a survey of microbiological laboratories, 11,167 cultures of coagulase-negative species were evaluated [21], whereby samples in which only one bottle was positive were interpreted as contamination in 75.2% of cases and samples in which two bottles were positive were counted as contamination in 27.8% of cases.

Another study assessed the likelihood of true bacteremia in combination with other clinical parameters and concluded that the positive predictive value for a true infection was 2% if only one of four bottles was positive, 9% if two bottles were positive, 13% if 3 bottles were positive and 27% if all four bottles were positive [35]. In conclusion, even with two positive findings of coagulase-negative staphylococci, the interpretation remains inconclusive, and contamination is more likely than true bacteremia if other clinical parameters are unremarkable. In the present study, the additional assessment of the antimicrobial susceptibility pattern was also conducted, which did not provide any indication of a congruent pathogen.

The significance of the TTP in differentiating between true bacteremia and contamination is a topic of controversy in the literature. In the presented study, the TTP was 45.5 ± 47.6 h in the study group and 22.6 ± 10.1 h in the control group, with no significant difference between the groups (*p* = 0.200). It is suggested that a higher inoculum of bacteria leads to faster organism growth [19,36,37,38]. Assuming that true bacteremia results in a higher inoculum of bacteria than contamination, Haimi-Cohen et al. noted in a pediatric study cohort that a TTP of ≤15 h is highly likely to indicate a true infection, whereas a TTP of ≥22 h can probably be considered as contamination [38]. However, given the comparatively high incidence of low-grade infections with a small bacterial load or non-virulent organism in the context of a PJI [18], this information should be taken into account within the PJI study cohort but evaluated with caution. Additionally, other authors have described no benefit of the TTP in differentiating between bacteremia and contamination [26,39].

In addition to the microbiological findings, histological investigations of the periprosthetic membranes were conducted according to the Krenn classification [40]. The sensitivity of this classification for detecting infections was reported as 73% and the specificity as 95% [41]. Only one patient with a positive ICS blood sample was classified as type II in terms of the infectious type. However, in this case, the initial PJI and the ICS microorganism were not congruent, and the patient showed no signs of persistent PJI or other septic complications during the follow-up.

Summarizing the assessment parameters described above, there was no evidence of persistent exposure of the ICS blood to PJI pathogens and no evidence of true bacteremia due to the microorganisms detected.

### 3.3. Follow-Up

In our study, no signs of septic complications were found in the immediate short-term, intermediate or long-term follow-up. In the scientific literature, the addition of clinical information is a common and well-established practice for interpreting inconclusive microbiological findings [19,22,38]. A recent study involving a larger number of cases also showed no increase in septic complications following the use of ICS during the reimplantation in two-stage revisions of septic hip arthroplasties [16].

### 3.4. Summary and Discussion of the Results in the Context of Pre-Existing Scientific Literature

In summary of the assessed parameters, including the type of organism detected, the antimicrobial susceptibility pattern, the number of positive bottles within a set, the TTP, the histological type according to Krenn and the clinical follow-up, there was no indication that the ICS blood from either the study or control group was colonized with a clinically relevant bacterial load. Above all, no persistence of the initial PJI microorganisms in the processed ICS blood was detected in the study cohort.

This is the first study in the orthopedic scientific literature to examine ICS blood in arthroplastic surgery in the context of PJIs. However, positive ICS cultures have also been reported in 67% to 70% of cases in the literature from other surgical areas, such as liver transplantation, cardiopulmonary bypass surgery or abdominal surgery [42,43]. The bacterial spectrum resembled that of the present study, with a high proportion of coagulase-negative staphylococci. Blood cultures taken from the patients one day postoperatively consistently yielded negative results. Likewise, the patients in the studies did not show an increase in septic complications; therefore, the use of ICS continues to be recommended for the described surgical fields.

This high rate of contamination in ICS blood can possibly be explained by the fact that microorganisms from the natural skin flora are present to a certain extent in deeper skin layers, and only approximately 80% of these organisms can be eradicated even with appropriate skin antisepsis [19,44,45]. Another explanation was provided by Ishida et al., who examined the bacterial spectrum in the ICS blood of patients undergoing open-heart surgery and compared it with the bacterial spectrum found on blood agar plates that had been placed next to the operating bed for 30 min [46]. A contamination rate of 82% was found in the ICS blood, with the bacteria observed being largely identical to those found on the blood agar plates, leading the authors to conclude that airborne contaminants were the main source of ICS contamination.

In summary, the aim of this study was to further evaluate the safety of ICS in the context of reimplantation during a septic two-stage hip revision surgery. The pathogens causing PJIs were no longer detectable in the processed ICS blood. Despite a high detection rate of other microorganisms in the ICS blood, this did not appear to be clinically relevant in relation to the assessed parameters described above. Therefore, ICS in the context of a reimplantation after septic two-stage hip revision surgery was considered a safe and recommendable procedure to reduce allogeneic pRBC transfusion and optimize patient blood management.

### 3.5. Limitations and Possible Future Research Perspectives

As with every study, certain limitations occur. For example, only the follow-up of patients who continuously returned for outpatient follow-up appointments could be examined. Furthermore, due to the study design in terms of an internal hospital quality control and the subsequent retrospective evaluation, only a small number of patients and no intervention was possible. Due to the small number of patients, it is only possible to generalize the findings to a limited extent. At the same time, possible research questions for future studies arise. For example, can the rate of positive ICS findings be influenced by the use of an incision foil or specific additional filter systems? In a larger patient population, it could also be investigated whether there is a correlation between the contamination rate of the ICS blood and the surgery duration, the blood loss, patient comorbidities or the respective surgeon. The answers to these questions could further contribute to optimizing the treatment of patients with PJIs.

## 4. Materials and Methods

### 4.1. Study Design and Patients

As part of the hospital’s internal quality control, blood culture samples were taken from the ICS blood of 12 patients during reimplantation following two-stage septic hip revision surgery to rule out the persistent presence of pathogens from the previous PJI (study group). Since pathogens with unclear clinical relevance were detected in 70% of the samples, additional samples were subsequently collected from 8 control patients during aseptic hip revision surgery (control group). In this group as well, microorganisms without clinical relevance were detected in 63% of the patients, leading to the decision to stop sampling to avoid unnecessary blood loss. The total number of patients involved in the study was 20.

Samples were collected between November 2020 and November 2021 and the patient’s follow-up was tracked until July 2024. Inclusion criteria for the study group were patients of all ages and sexes who underwent reimplantation, as sufficient infection treatment was assumed. Inclusion criteria for the control group were patients of all ages and sexes undergoing aseptic hip revision due to implant loosening. In both groups, the blood loss had to be high enough for ICS blood to be processed. No further exclusion criteria were defined. A retrospective evaluation of the resulting data was conducted.

The usual clinical routines were carried out without changes. In this context, pre-operative percutaneous joint needle aspiration was performed on all patients prior to planned revision surgery. During each revision procedure, whether septic or aseptic, five tissue samples were taken intraoperatively for microbiological diagnostics and one tissue sample for histological investigation. Each microbiological sample was incubated for 14 days.

The standard antiseptic measures included an antiseptic body wash, bowel cleansing and shaving of the surgical site on the evening before surgery. Preoperative antibiotics were administered in both groups 30 to 60 min before skin incision. Patients in the reimplantation group (study group) received antibiotics that matched the resistogram of the pathogen causing PJI, while patients in the aseptic revision group (control group) received a prophylactic dose of cefazolin or clindamycin in case of allergy. Sterile washing and draping were performed in accordance with standard hygienic guidelines.

The ICS samples were collected under the following strictly sterile conditions: a separate sterile table was prepared for sample processing. The sampling and inoculation of the blood culture bottles were performed using sterile gloves. In each case, 2 × 10 mL of ICS blood was drawn from the dedicated device and one aerobic and one anaerobic blood culture bottle were inoculated. The blood culture bottles were immediately sent to the processing laboratory, which then began the incubation process.

The following data were collected and analyzed retrospectively: patient and surgical characteristics; results of all microbiological samples obtained from preoperative joint needle aspirates, intraoperative tissue samples or ICS blood; the histological classification of the periprosthetic membranes according to Krenn [40]; as well as immediate postoperative follow-up, intermediate follow-up with observation of inpatient stay and subsequent rehabilitation and long-term follow-up with all outpatient re-presentations recorded during the course.

The collected data were documented anonymously in an Excel file (version 16.72, Excel for Mac, Microsoft, Redmond, Washington, United States) and saved on a password-protected computer.

### 4.2. Statistical Analysis

The data analysis was performed using both descriptive and inferential statistical methods using the statistical program SPSS 29.0 (SPSS, Chicago, IL, USA). This combined approach enabled the summarization of the data and facilitated the derivation of meaningful conclusions from the findings. Continuous variables were expressed as mean ± standard deviation (SD) to indicate variability while categorical variables were expressed as number and percentage (%). Group comparisons were conducted using *t*-tests for independent variables for normally distributed continuous variables and Mann–Whitney U tests for non-normally distributed variables. The Shapiro–Wilk normality test was applied to assess the normality of the data distribution, allowing informed decision on the appropriateness of parametric or non-parametric tests. Categorical variables were analyzed using the Fisher’s exact tests, which is particularly suitable for small sample sizes and 2 × 2 contingency tables with low expected frequencies. This approach ensures the reliability of the results by providing an exact *p*-value under these conditions. A significance level of 0.05 was set for all statistical analyses, with *p*-values below this threshold considered statistically significant.

## 5. Conclusions

In the context of reimplantation following a septic two-stage hip revision surgery, the initial pathogens responsible for the PJI were no longer detectable in the processed ICS blood. A high contamination rate of the ICS samples was observed; however, this did not show any clinical relevance. ICS during reimplantation was considered a safe technique to reduce allogeneic pRBC transfusions and optimize patient blood management, but further research is recommended to continue evaluating the safety of the procedure.

## Figures and Tables

**Table 1:** Patient and surgery characteristics, ICS cultures and possible signs of sepsis of the study and control groups.

Patient and Surgery Characteristics	Total n = 20	Study Group n = 12	Control Group n = 8	*p*-Value ***
Age (yr) *	71.4 ± 9.9	70.8 ± 8.3	72.4 ± 12.5	0.757
Male sex **	10 (50%)	5 (42%)	5 (63%)	0.650
BMI (kg/m^2^) *	29.8 ± 7.1	29.6 ± 8.5	30.1 ± 4.7	0.440
ASA score >2 **	12 (60%)	7 (60%)	5 (63%)	0.852
Surgery duration (min) *	202 ± 76	195 ± 72	211 ± 85	0.643
Estimated blood loss (mL) *	1397 ± 528	1265 ± 367	1595 ± 684	0.330
Volume of transfused ICS blood (mL) *	403 ± 151	360 ± 85	467 ± 207	0.330
Total number of allogeneic blood units transfused *	1.84 ± 2.2	2.75 ± 2.2	0.75 ± 1.5	**0.022**
**ICS blood culture results**				
Positive ICS blood cultures **	14 (70%)	9 (75%)	5 (63%)	0.642
Time to positivity (h) *	34.1 ± 35.1	45.5 ± 47.6	22.6 ± 10.1	0.200
**Possible signs of sepsis**				
Leucocytes pre-op. (G/L) *	6.6 ± 1.8	6.3 ± 1.6	6.9 ± 2.2	0.440
Leucocytes 2 days post-op. (G/l) *	7.6 ± 1.9	7.2 ± 1.4	8.2 ± 2.5	0.334
Mean arterial pressure (MAP) 2 days post-op. (mmHg) *	90 ± 11	90 ± 13	90 ± 9	0.877
Body temperature 2 days post-op. (°C) *	36.8 ± 0.5	36.9 ± 0.6	36.8 ± 0.4	>0.999

* Presentation of the values as mean and standard deviation. ** Presentation of the values as total amount and percentage. *** *p*-values assessed by Fisher’s exact test or Mann–Whitney U test, as appropriate.

**Table 2:** Type of pathogen found in the positive ICS blood cultures categorized by study and control group.

	Total n = 14/20 (70%)	Study Group n = 9/12 (75%)	Control Group n = 5/8 (63%)
*Staph. epidermidis*	6	4	2
*Staph. capitis*	2	0	2
*Cutibac. acnes*	2	1	1
*Candida albicans*	2	2	0
*Staph. hominis*	1	0	1
*Staph. aureus*	1	1	0
*Staph. haemolyticus*	1	1	0
*Gram-positive bacilli*	1	1	0

**Table 3:** Krenn classification of the periprosthetic membranes, subdivided into patients with positive and negative ICS cultures.

	Total n = 20	Positive ICS Culture n = 14	Negative ICS Culture n = 6
**I** (particle type)	7 (35%)	2 (%)	5 (%)
**II** (infectious type)	1 (5%)	1 (%)	0 (%)
**III** (combined type)	2 (10%)	2 (%)	0 (%)
**IV** (indifferent type)	6 (30%)	5 (%)	1 (%)
**not identified**	4 (20%)	4 (%)	0 (%)

## Data Availability

Data are available from the authors on reasonable request.
